# Long-term effects on fertility after central nervous system cancer: A systematic review and meta-analysis

**DOI:** 10.1093/nop/npae078

**Published:** 2024-08-29

**Authors:** Janna Pape, Tanya Gudzheva, Danijela Beeler, Susanna Weidlinger, Angela Vidal, Rhoikos Furtwängler, Tanya Karrer, Michael von Wolff

**Affiliations:** Division of Gynecological Endocrinology and Reproductive Medicine, University Women´s Hospital, Inselspital Bern, University of Bern, Bern, Switzerland; Division of Gynecological Endocrinology and Reproductive Medicine, University Women´s Hospital, Inselspital Bern, University of Bern, Bern, Switzerland; Division of Gynecological Endocrinology and Reproductive Medicine, University Women´s Hospital, Inselspital Bern, University of Bern, Bern, Switzerland; Division of Gynecological Endocrinology and Reproductive Medicine, University Women´s Hospital, Inselspital Bern, University of Bern, Bern, Switzerland; Division of Gynecological Endocrinology and Reproductive Medicine, University Women´s Hospital, Inselspital Bern, University of Bern, Bern, Switzerland; Division of Pediatric Hematology and Oncology, Department Of Pediatrics, Inselspital, University of Bern, Bern, Switzerland; Medical Library, University Library Bern, University of Bern, Bern, Switzerland; Division of Gynecological Endocrinology and Reproductive Medicine, University Women´s Hospital, Inselspital Bern, University of Bern, Bern, Switzerland

**Keywords:** cancer, central nervous system, fertility, fertility preservation, FertiPROTECT, FertiTOX, gonadotoxicity

## Abstract

**Background:**

Central nervous system (CNS) cancer represents a common group of solid tumors in childhood and young adults, and less frequently in adults aged 30–40. Due to treatment advancements with increasing survival rates, disorders of the hypothalamus-pituitary axis have become increasingly relevant for patients’ future fertility plans. Most guidelines recommend that physicians should counsel their patients about fertility prognosis before initiating gonadotoxic therapy. However, for fertility preservation measures, gonadal toxicity as the only relevant risk factor has not yet been systematically reviewed.

**Methods:**

A systematic literature search was performed in MEDLINE, Embase, and Cochrane in January 2024. The systematic review included studies of patients who had undergone treatment for all types of malignant CNS cancer. The outcomes were defined as clinically relevant gonadal toxicity as well as preserved fertility. The study adheres to the Preferred Reporting Items for Systematic Reviews and Meta-Analysis (PRISMA) guidelines.

**Results:**

The qualitative analysis included 31 studies with a total of 4590 patients after CNS cancer. The overall pooled prevalence of gonadal toxicity was found to be 20% (95% confidence intervals [CI]: 10%–34%). Preserved fertility was present in 75% (95% CI: 64%–83%) of the patients and was maintained after at least 5 years following treatment (75%, 95% CI: 46%–91%).

**Conclusions:**

This initial meta-analysis provides a basis for fertility counseling after diverse CNS cancer treatments. Due to the high heterogeneity of the study population and lack of individual patient data on fertility outcomes, it is not possible to provide an exact estimation of the fertility prognosis following a specific treatment. Thus, fertility preservation measures should still be recommended.

Central nervous system (CNS) cancer is the most common group of solid tumors in childhood and adolescence, and the leading cause of cancer-related mortality in this age group.^1,2^ CNS tumors occur less frequently in adults between 30 and 40 years of age,^1^ yet affect a demographic where family planning is often not yet complete. Due to advancements in treatment modalities, the survival rate in CNS cancer survivors is increasing, so that endocrine disorders and fertility issues may be encountered more frequently.^3,4^

Complications following oncological treatments using alkylating agents and radiotherapy often include impairment in gonadal function.^5,6^ Cranial irradiation damages the hypothalamic-pituitary axis (HPG), while chemotherapy can have direct gonadotoxic effects. The cumulative dosage of chemotherapeutic agents and radiotherapy can impair fertility through both direct gonadotoxic effects and impairment of the hypothalamic-pituitary-gonadal (HPG) axis.^7,8^

Infertility can have significant effects on the psychological and emotional well-being of cancer survivors; hence, fertility preservation is now considered the gold standard before initiating cancer treatment. Current guidelines suggest that physicians should advise their patients on fertility preservation measures before starting gonadotoxic therapy.^9–11^ Fertility preservation options prior to gonadotoxic exposure are well-established for patients who have undergone puberty. However, options for prepubertal patients who are unable to produce mature gametes are limited and include cryopreservation of ovarian and testicular tissue, which is still experimental before the onset of puberty.^12^ Given that fertility preservation measures can carry medical risks and burdens for patients, as well as potentially delay cancer therapies, it is essential to assess the risk of infertility resulting from the gonadal toxicity of cancer treatment.

So far, few studies have evaluated fertility in CNS cancer patients following various oncological treatments. Case reports have been published of CNS cancer survivors who have produced healthy children after treatment with temozolomide and radiotherapy. However, no large population-based studies have been conducted.^13,14^

Given the limited longitudinal data available, this systematic review with meta-analysis aims to provide an approximation of gonadal toxicity and preserved fertility after CNS treatments for fertility counseling.

We have initiated a series of systematic reviews to establish a literature platform on the gonadal toxicity of different cancer group-specific therapies.^15–17^ This series is part of the project FertiTOX (www.fertitox.com) which also involves a prospective international multicenter data collection on the gonadal toxicity of cancer therapies in females and males.^18^

## Materials and Methods

### Protocol Registration

The study protocol was registered at the International Prospective Register of Systematic Reviews, PROSPERO (Registry number CRD42023385408). The Preferred Reporting Items for Systematic Reviews and Meta-Analysis (PRISMA) were used.

### Search Strategy

A systematic literature search was conducted in Embase via Ovid, MEDLINE ALL via Ovid and the Cochrane Database of Systematic Reviews, and the Cochrane Central Register of Controlled Trials via Wiley. The original search was performed in December 2022, and last updated on January 24th, 2024. An initial search strategy was developed in Embase by a medical information specialist and tested against a list of core references. After refinement and consultation, comprehensive search strategies were set up for each information source based on database-specific controlled vocabulary (thesaurus terms / subject headings) and text words. Synonyms, acronyms, and similar terms were included in the text word search. The search was limited to publications since 2000. The search concepts included all types of CNS cancer, oncological therapies, and gonadotoxic effects reflected by influences on fertility parameters. The MEDLINE and Embase searches excluded animal-only studies using a double-negative search strategy based on the “humans only” filters by Ovid. The detailed final search strategies are presented as Supplementary Figure S1. Reference lists and bibliographies were scanned for relevant studies. References were imported into Deduklick^19^ and duplicates were removed.

### Inclusion and Exclusion Criteria

The studies were assessed for inclusion by 3 investigators (TG, DB, and JP) using the Covidence software (www.covidence.org). Original papers containing information on tumor type, tumor therapy, and fertility results with numerical data enabling prevalence calculation of gonadal toxicity and/or preserved fertility were considered eligible. All types of high-grade/malignant and low-grade CNS tumors were included.

Gonadal toxicity was defined as basal LH or FSH levels above the upper limit of the reference range and/or low AMH levels in women or low inhibin B in men, and/or azoo-/oligospermia. Preserved fertility was indicated by the absence of signs of gonadal toxicity including primary/secondary amenorrhea, no central/primary/secondary hypogonadism, or panhypopituitarism.

Studies on (benign) pituitary tumors were excluded.

### Data Extraction

Three investigators (JF, DM, and JP) independently abstracted and reviewed the extracted data in detail. Key variables of interest were: Characteristics of the study populations (age of patients at diagnosis of CNS tumor and outcome, length of follow up, and ethnicity), histology of CNS tumor, oncological treatment (types and dosages of chemo- and radiotherapy, combined therapies), and fertility parameters. Discrepancies were discussed and resolved by consensus.

### Quality Assessment

The quality of the studies was assessed using the Newcastle-Ottawa Scale. Each study was scored based on 3 parameters: Subject selection (0–4 stars), comparability (0–2 stars), and study outcome (0–3 stars). The final rating was determined by the number of stars in the selection, comparability, and outcome/exposure domains, classifying the study quality as good, fair, or poor. Studies were rated based on quality, with good quality studies receiving 3 or 4 stars in the selection domain, 1 or 2 stars in the comparability domain, and 2 or 3 stars in the outcome/exposure domain. Fair quality studies received 2 stars in the selection domain, 1 or 2 stars in the comparability domain, and 2 or 3 stars in the outcome/exposure domain. Poor quality studies received 0 or 1 star in the selection domain, 0 stars in the comparability domain, or 0 or 1 stars in the outcome/exposure domain.

TG, DB, and JP independently assessed the risk of bias, and any disagreements were resolved by consensus.

### Data Synthesis

The systematic review aimed to determine the prevalence of gonadal toxicity and preserved fertility in patients who underwent oncological treatments for CNS cancer. Gonadal toxicity prevalence was calculated by dividing the number of patients who met the criteria for gonadal toxicity and/or preserved fertility (compare inclusion criteria) by the number of patients at risk for the outcomes in each study. The metafor function in R software (R Core Team, Vienna, Austria, 2013) was used to analyze the pooled prevalence. To assess heterogeneity, we used Cohen’s Q statistic and I^2^ statistic. In cases of high heterogeneity, random effects models were employed. We excluded studies that included women who had undergone ovarian tissue transplantation (as ovarian function is affected by tissue removal and freezing as well as by tissue transplantation).

## Results

### Systematic Review Results

**Figure 1.**
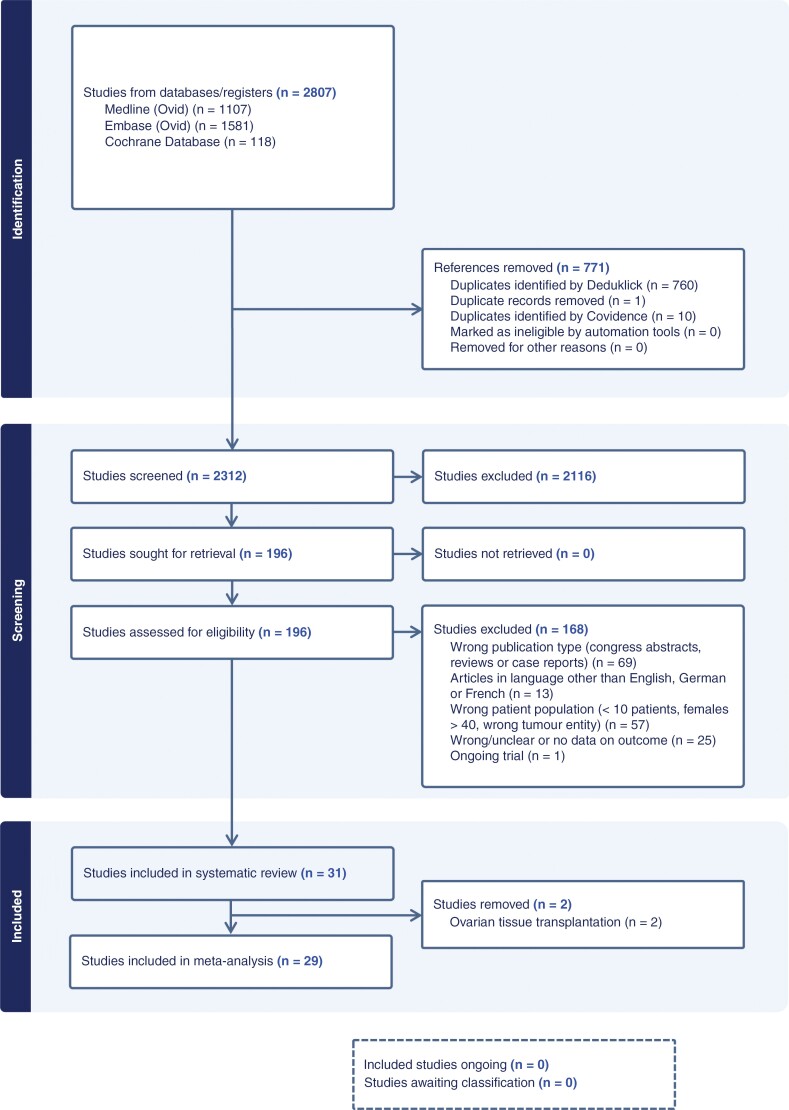
Preferred Reporting Items for Systematic Reviews and Meta-Analysis *(PRISMA) flow diagram.* Flowchart of the literature search and selection process.

After screening the abstracts and full texts, 196 studies were considered. Of these, 168 studies did not meet the criteria and were excluded. Finally, 31 articles met our inclusion criteria and were included in the systematic review (Figure 1).

#### Study characteristics.—

Characteristics of the study populations are summarized in Supplementary Figure S2. The analysis included retrospective (*n* = 17), prospective (*n* = 6), cross-sectional (*n* = 3), and case–control studies (*n* = 3), as well as 2 patient surveys. The majority of the studies were rated as poor (*n* = 14) or fair (*n* = 12) in terms of methodological quality. This was primarily due to the absence of a comparison group, small sample sizes, short follow-up periods, or the failure to evaluate pretreatment endocrinopathies (Table 1).

**Table 1.** *Bias Screening* Newcastle-Ottawa Quality Assessment Form for Cohort Studies

	Selection				Comparability		Outcome					
Study, year	Representativeness of exposed cohort	Selection of non-exposed cohort	Ascertainment of exposure	Outcome of interest not present at start of study	Comparability of cohorts in main factors	Comparability of cohorts in additional factors	Assessment of outcome	Sufficient length of follow-up for outcomes to occur	Adequacy of follow-up of cohorts	Total	Quality Assessment	Comments
Saeki, 2000	**★**	—	**★**	—	**—**	—	**—**	**★**	**★**	4/9	Poor	small study size, old data
Gurney, 2003	**★**	**★**	**★**	—	**★**	—	**—**	**★**	**★**	6/9	Good	siblings as comparative group
Agha, 2005	**★**	**★**	**★**	—	**★**	—	**★**	**★**	**★**	7/9	Good	adequate control group (no radiotherapy)
Cuny, 2011	**★**	—	**★**	—	**★**	—	**★**	**—**	**★**	5/9	Poor	time of follow-up not described
Madaschi, 2011	**★**	—	**★**	—	**—**	—	**★**	**★**	**—**	4/9	Poor	inadequate control group and follow-up
Preusser, 2011	**★**	—	**★**	—	**—**	—	**★**	**★**	**★**	5/9	Poor	cross-sectional study, small study size
Shlomit, 2011	**★**	—	**★**	—	**—**	—	**★**	**★**	**★**	5/9	Poor	pretreatment endocrinopathies not evaluated
Viswanathan, 2011	**★**	—	**★**	—	**—**	—	**★**	**—**	**★**	4/9	Poor	short follow up
Koustenis, 2013	**★**	—	**★**	**★**	**—**	—	**—**	**★**	**★**	5/9	Poor	survey
DeWire, 2014	**★**	—	**★**	**—**	**★**	—	**★**	**★**	**★**	6/9	Fair	pretreatment endocrinopathies not evaluated
Balachandar, 2015	**★**	—	**★**	**—**	**★**	—	**★**	**★**	**★**	6/9	Fair	pretreatment endocrinopathies not evaluated
Pfitzer, 2014	**★**	—	**★**	**—**	**—**	—	**—**	**★**	**—**	3/9	Poor	poor registry data
Shih, 2014	**★**	—	**★**	**★**	**—**	—	**★**	**★**	**★**	5/9	Poor	small study size
Uday, 2015	**★**	—	**★**	**—**	**★**	—	**★**	**★**	**★**	6/9	Fair	small study size
Pietila, 2017	**★**	—	**★**	—	**—**	—	**★**	**★**	**★**	5/9	Poor	cross-sectional study
Vatner, 2018	**★**	—	**★**	**★**	**★**	—	**★**	**★**	**★**	7/9	Good	baseline and detailed outcome evaluation
Jalali, 2019	**★**	—	**★**	**★**	**★**	—	**★**	**★**	**★**	7/9	Good	detailed outcome evaluation
Santos, 2019	**★**	—	**★**	**—**	**—**	—	**★**	**★**	**★**	5/9	Poor	pretreatment endocrinopathies not evaluated
Van Iersel, 2020	**★**	—	**★**	—	**★**	—	**★**	**★**	—	5/9	Fair	pretreatment endocrinopathies not evaluated
Xiang, 2020	**★**	—	**★**	**—**	**—**	—	**★**	**—**	**—**	3/9	Poor	short follow up
Haghiri, 2021	**★**	—	**★**	**—**	**★**	—	**—**	**★**	**★**	5/9	Poor	pretreatment endocrinopathies not evaluated
Maciel, 2021	**★**	—	**★**	**—**	**★**	—	**★**	**★**	**★**	6/9	Fair	pretreatment endocrinopathies not evaluated
Zhang, 2021	**★**	—	**★**	—	**★**	—	**★**	**★**	—	5/9	Fair	high percentage of loss to follow-up
Gonzales, 2022	**★**	—	**★**	**—**	**★**	—	**★**	**★**	**—**	5/9	Fair	pretreatment endocrinopathies not evaluated
Margolis, 2022	**★**	—	**★**	**—**	**★**	—	**★**	**★**	**★**	6/9	Fair	pretreatment endocrinopathies not evaluated
Merchant, 2022	**★**	—	**★**	**★**	**★**	—	**★**	**★**	**★**	7/9	Good	baseline and detailed outcome evaluation
Partenope, 2022	**★**	—	**★**	—	**—**	—	**★**	**★**	**★**	5/9	Poor	pretreatment endocrinopathies not evaluated
Abali, 2023	**★**	**—**	**★**	**—**	**★**	—	**★**	**★**	**★**	6/9	Fair	pretreatment endocrinopathies not evaluated
Merchant, 2023	**★**	**—**	**★**	**—**	**★**	**—**	**★**	**★**	**★**	7/9	Fair	pretreatment endocrinopathies not evaluated
Rosimont, 2023	**★**	—	**★**	**—**	**★**	—	**★**	**★**	**★**	6/9	Fair	pretreatment endocrinopathies not evaluated
Stern, 2023	**★**	—	**★**	—	**★**	—	**★**	**★**	**★**	6/9	Fair	pretreatment endocrinopathies not evaluated

Newcastle-Ottawa Quality Assessment Form for Cohort Studies.

A total of 4590 patients reported a history of CNS cancer, of which 3854 (84.0%) were eligible for fertility analysis. The sample sizes of the studies ranged from 11 to 1607 patients. Sixteen studies reported on gonadal toxicity, while all others evaluated effects on the HPG axis.

The studies were conducted in various regions, including Europe (*n* = 14), Asia (*n* = 5), and the USA (*n* = 12). The CNS tumors’ histology comprised gliomas (39.3%), medulloblastomas (26.3%), ependymal (10.2%), germ cells (7.1%), and sellar tumors (4.1%).

The study participants were primarily prepubertal, with a mean age of 11.5 years (range 2.6–38.5) at the time of cancer diagnosis and 17.1 years (range 3–39.3) at the time of outcome evaluation. The studies had a lengthy follow-up period, averaging 8.9 years and ranging from 1.6 to 25 years. The treatment options included surgery (80.5%), various protocols of chemotherapy (48.1%) including high-dose chemotherapy (20.3%), stem cell transplantation (4.5%), and/or different dosages and types of radiotherapy (62.5%). Patients who received cranial (26.3%) or craniospinal (14.4%) radiotherapy were treated with either photon (69.6%) or proton (5.5%) radiotherapy, while the type of radiotherapy was not specified in 59.3% of the patients. In the studies which reported the numbers of patients per individual therapy, treatments comprised only surgery (6.23%) or combinations with radiotherapy (35.4%), chemotherapy (12.5%), or both (32.3%).

#### Prevalence of gonadal toxicity.—

The prevalence of gonadal toxicity in patients with a history of CNS cancer and oncological treatment varies widely, ranging from 2.6% to 83.8%. In a study that included patients after autologous stem cell transplantation, the highest prevalence of gonadal toxicity was reported at 83.8%.^20^ Retrospective studies of long-term survivors (mean follow-up 12.8 years) with childhood brain tumors^21^ and of prepubertal patients with optic tract gliomas^22^ reported the lowest prevalences at 2.6% and 3.8%, respectively.

#### Prevalence of preserved fertility.—

The reported prevalence of preserved fertility after CNS cancer treatments was reported to be generally high, ranging around 80%. Lowest rates of preserved fertility are reported in studies with patients after short follow-up,^23–26^ after stem cell transplantation^4,20^ or in high-risk patients^27^ Three studies reported outcome data separately for prepubertal patients: A retrospective study in a cohort with optic tract gliomas showed a prevalence of preserved fertility of 86.5%.^22^ Another retrospective study^4^ including patients after stem cell transplantation reported a prevalence of preserved fertility of 66.1%, whereas the case–control study by Zhang et al.^26^ described the recovery of the HPG axis in only 26.7% (12/45); however, the follow-up was shorter than 5 years. For modern radiotherapy techniques, 3 studies evaluated fertility after proton-beam therapy and showed high rates of fertility preservation ranging from 87% to 95%.^28–30^

### Meta-Analysis Results

To make a valid statement on fertility outcomes due to standard therapies in patients with CNS tumors, 2 studies^4,31^ with patients after ovarian tissue transplantation before treatment were excluded. (Figure 1).

#### Pooled overall prevalence of gonadal toxicity.—

**Figure 2.**
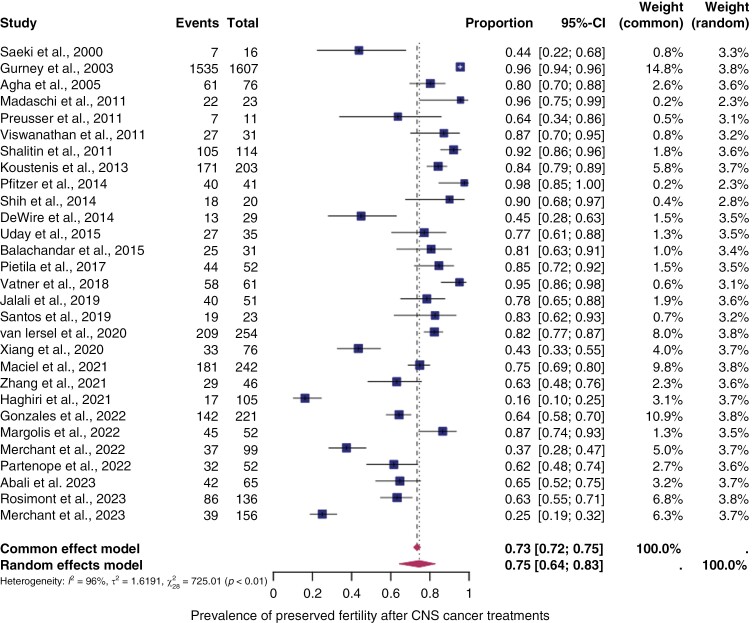
*The pooled overall prevalence of gonadal* toxicity. Forest plot of proportions and 95% confidence intervals (CI) for studies evaluating the prevalence of gonadal toxicity after central nervous system cancer treatments. Gonadal toxicity was defined as basal LH or FSH levels above the upper limit of the reference range and/or low AMH levels in women or low inhibin B in men, and/or azoo-/oligospermia. Squares for each study indicate the proportion, the size of the boxes indicates the weight of the study, and the horizontal lines indicate the 95% CI. The data in bold and diamond represent the pooled prevalence for post-treatment infertility and 95% CI. Overall estimates are shown in the fixed- and random-effect models.

Fourteen studies were included in the analysis of the overall prevalence of gonadal toxicity in CNS cancer therapy. These studies involved patients who underwent various oncological treatments, such as different types and dosages of chemotherapy, radiotherapy (including radiation of the hypothalamus and spine), and combinations of different therapies. Figure 2 displays the prevalence for each study and the summary prevalence. The overall prevalence of gonadal toxicity was found to be 14% (95% confidence intervals [CI]: 10%–34%). Significant heterogeneity was observed between the studies (I^2^ = 94%, *P* < .01).

#### The pooled overall prevalence of preserved fertility.—

**Figure 3.**
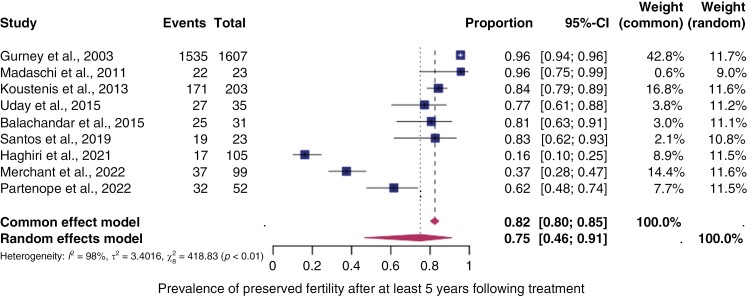
*Pooled prevalence of preserved fertility.* Forest plot of proportions and 95% confidence intervals (CI) for studies evaluating the prevalence of preserved fertility after central nervous system cancer treatments. Preserved fertility was defined as the absence of gonadal toxicity including primary/secondary amenorrhea, no central/primary/secondary hypogonadism, or panhypopituitarism. Squares for each study indicate the proportion, the size of the boxes indicates the weight of the study, and the horizontal lines indicate the 95% CI. The data in bold and diamond represent the pooled prevalence for post-treatment infertility in good prognosis patients and 95% CI. Overall estimates are shown in the fixed- and random-effect models.

All of the included studies in the meta-analysis reported effects on the HPG-axis, enabling calculation of the prevalence of preserved fertility. Figure 3 shows the prevalence of preserved fertility for each study and the summary prevalence. The overall prevalence in patients after CNS cancer treatment was 75% (95% CI: 64%–83%), with significant heterogeneity between the studies (I^2^ = 96%, *P* < .01).

**Figure 4.**
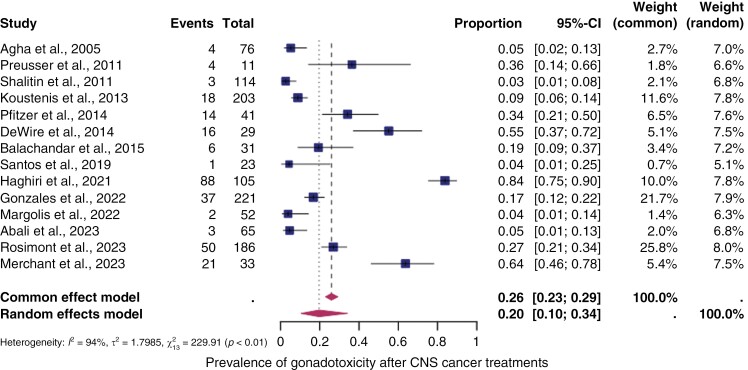
*The pooled prevalence of preserved fertility after at least 5 years following treatment.* Forest plot of proportions and 95% confidence intervals (CI) for studies evaluating the prevalence of preserved fertility in central nervous system cancer survivors after at least 5 years following treatment. Preserved fertility was defined as the absence of gonadal toxicity including primary/secondary amenorrhea, no central/primary/secondary hypogonadism, or panhypopituitarism. Squares for each study indicate the proportion, the size of the boxes indicates the weight of the study, and the horizontal lines indicate the 95% CI. The data in bold and diamond represent the pooled prevalence for post-treatment infertility in good-prognosis patients and 95% CI. Overall estimates are shown in the fixed- and random-effect models.

A subgroup analysis was conducted on studies that only included patients with a follow-up of at least 5 years. The analysis showed a similar prevalence of preserved fertility at 75% (95% CI: 46%–91%) as depicted in Figure 4.

**Figure 5.**
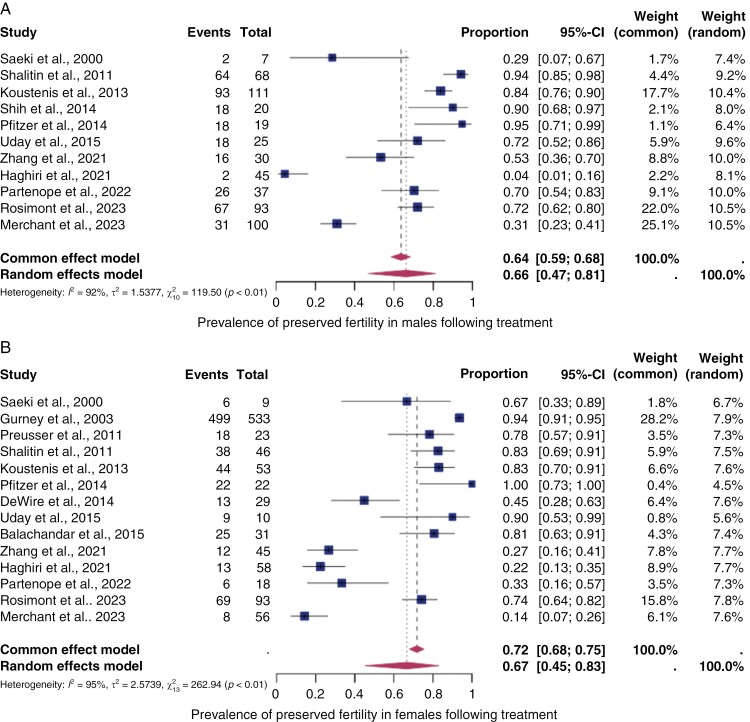
*The pooled prevalence of preserved fertility by sex.* Forest plot of proportions and 95% confidence intervals (CI) for studies evaluating the prevalence of preserved fertility in male **(A)** and female **(B)** central nervous system cancer survivors. Preserved fertility was defined as the absence of gonadal toxicity including primary/secondary amenorrhea, no central/primary/secondary hypogonadism, or panhypopituitarism. Squares for each study indicate the proportion, the size of the boxes indicates the weight of the study, and the horizontal lines indicate the 95% CI. The data in bold and diamond represent the pooled prevalence for post-treatment infertility in good prognosis patients and 95% CI. Overall estimates are shown in the fixed- and random-effect models.

Eleven studies reported outcome data by sex revealing a pooled prevalence of 74% (95% CI: 60%–84%) in males and 67% (95% CI: 45%–83%) in females (Figure 5).

## Discussion

The aim of this systematic review and meta-analysis was to analyze fertility outcomes in CNS cancer survivors to improve counseling on fertility preservation. This is the first meta-analysis to assess the pooled prevalence of fertility outcomes. As treatment in CNS cancer comprises effects both on the gonads and on the HPG axis, and as the indication for fertility preservation is only based on gonadal toxicity, we evaluated gonadal function and preserved fertility separately.

Our study revealed the following important findings in the general population of CNS cancer:

First, the overall pooled prevalence of gonadal toxicity in the general population of CNS cancer survivors is moderate (20%, 95% CI: 10%–34%).

Second, the overall pooled prevalence of preserved fertility is relatively high (75%, 95% CI: 64%–83%) and comparable between males and females (males: 67% [95% CI: 45%–83%]; females: 67%, 95% CI: 45%–83%).

Third, the prevalence of preserved fertility is maintained after at least 5 years following treatment (75%, 95% CI: 46%–91%).

However, the quality of studies on this topic is low, despite its high clinical relevance. There were 5 studies of good quality and 12 studies of fair quality. Spermiograms (ie, evaluation of sperm quality with regard to concentration and motility) were performed after treatments in 2 single studies.^27,32^ Blood parameters for fertility (ie, LH/FSH, AMH, and inhibin) without only direct interpretation (ie, hypo- or hypergonadotropic hypogonadism, gonadal failure) were explicitly reported in 6 studies.^20,25,27,28,33,34^

The pooled prevalence of gonadal toxicity in our study is higher than that reported in some of the included studies.^22,35,36^ This difference may be due to heterogeneous study cohorts with different types of cancer and therapy, methodological weaknesses in the individual studies, length of follow-up, definitions of hypothalamic-pituitary-gonadal axis (HPGA) dysfunction, and timing and protocol of endocrine evaluation. On the other hand, a higher prevalence of gonadal toxicity is described in studies with reported measurements of follicle-stimulating hormone (FSH)/luteinising hormone (LH) and/or anti-Mullerian hormone (AMH).^25,27,37,38^ Only one study^4^ defined a low AMH value as being lower than the reference range. The other 3 studies^27,31,37^ did not provide a definition for low values. Additionally, various enzyme-linked immunosorbent assays (ELISA) were used globally for AMH measurements until 2015, which hampers the comparability.^39^

Our study found a higher pooled prevalence of preserved fertility compared to the largest of the more recent studies by Gonzales et al.,^33^ which reported a prevalence of 64% (95% CI: 58%–70%). In this prospective study, the criteria for gonadal toxicity were clearly defined and strictly applied which is in contrast to the majority of the included studies.

In almost all of the studies included the analysis was limited to prepubertal or young postpubertal patients. Only 2 studies examined older populations over the age of thirty, which demonstrated similar^35^ or lower^25^ rates of preserved fertility.

The prevalence of preserved fertility in the pooled cohort of our study was comparable between males and females; however, no clear statement can be made about sex differences due to the lack of homogeneity of the cohorts. Additionally, spermiograms were conducted on only a small number of patients following treatment. In literature, it has been shown that a single dose of radiation damages the germinal epithelium in the testes to a lesser extent than the same dose divided into fractions, while the opposite effect occurs in the ovaries.^40^

Further subgroup analyses by tumor type and location were not possible due to the limited number of cases, aggregated fertility outcomes of heterogeneous study cohorts, and the lack of individual patient data.

As treatment of CNS cancer can lead to both direct gonadotoxic effects and additional impairments of the HPGA, we analyzed both gonadotoxicity alone and the preservation of the HPGA assuming preserved fertility. This information is relevant for patients undergoing counseling as the direct effects of gonadotoxicity can make it difficult or even impossible to undergo reproductive medical treatment with their own gametes. However, reproductive medicine may still be possible if the gonads remain intact.

In our meta-analysis, the long-term effects on fertility following CNS cancer therapy appear to be moderate, so the need for fertility preservation should be discussed. In prepubertal patients, the risk of fertility preservation measures (ie, ovarian or testicular tissue cryopreservation) needs to be balanced against the risk of infertility. Fertility preservation is not universally available and, in some cases, its costs may not be fully covered by insurance, depending on the country’s legislation and the perceived risk of infertility.^41^ In relation to the utilization of cryopreserved reproductive material, the utilization rates for cryopreserved semen varied between 2.6% and 21.5%, for oocytes between 3.1% and 8.7%, and for embryos approximately 9% to 22.4%.^42^ Still, in adult patients with open family planning in the near future, we recommend oocyte and sperm cryopreservation before oncological treatment due to the uncertainty surrounding post-surgery therapy and the lack of comprehensive longitudinal data on individual treatment effects.

The available data on pregnancies following CNS tumors is limited. In the study by Iersel et al.,^34^ only 6.1% of pregnancies were described. One potential explanation might be the young age of cancer diagnosis, which means that patients have to be followed up for decades. Even in retrospective studies, some patients are not yet of family planning age at the time of evaluation. However, due to the paucity of data, it cannot be ruled out that the low pregnancy rates reflect reduced fertility following cancer treatment.

Even though our study strictly followed the recommendations to provide high-quality summary reports of evidence, some limitations are evident:

First, the majority of the included studies were based on retrospective or registry data with missing data on treatment protocols (ie, radiation dosimetry to the hypothalamus and spine, cumulative dose, and exact type of chemotherapy), as important risk factors for long-term fertility outcomes. Additionally, there were no studies from Africa, Australia, and South America, so no statement can be made for patients of these ethnic groups.

Second, dysfunction of the HPGA may have been present prior to oncological treatment, which could lead to an underestimation of the prevalence of preserved fertility, especially in patients with a short follow-up period. Only a few studies have evaluated endocrinopathies before treatment (Supplementary Figure S2).

Third, the aggregation of outcome data from different tumor types (i.a. gliomas of all grades) and treatments makes it difficult to accurately estimate fertility outcomes depending on the individual patient´s characteristics. The recent introduction of proton-beam irradiation^43^ and molecular-matched targeted therapies, many of them still in early-phase clinical trials,^44^ may also have an impact on fertility outcomes.

Fertility and, in particular, outcomes in the longer term, such as pregnancies after carcinoma therapy, were hardly analyzed in older studies. It should be noted that patients were often treated several years or decades before 2000. Treatments have been modernized since then, and at the time of cancer treatment, the majority of patients did not have access to the current intensity-modulated photon irradiation or proton radiotherapy. Two studies^28,30^ included patients treated with proton radiotherapy alone and showed fertility preservation rates >90%. This is consistent with the steep dose drop dorsal to the target volume in proton radiotherapy compared to current intensity-modulated photon irradiation.^45,46^ Nevertheless, only 3 papers published prior to 2010^24,35,47^ were incorporated into our meta-analysis, ensuring the continued relevance of our findings for contemporary patients. Following the year 2010, 3 studies^30,48,49^ were rated as being of good quality.

Although targeted therapies have been introduced in first-line treatments for childhood, adolescent, and young adult cancers, the majority of treatment approaches still rely on classical cytotoxic drugs. Current treatment optimization studies continue to use clinical risk stratification to reduce treatment where possible.^50^ Although “targeted therapy” implies a more specific effect on cells than conventional cytotoxic drugs, there are well-documented off-target effects, including fertility issues in preclinical models. Currently, there is a lack of reliable data on whether fertility and gonadotoxicity are a concern. However, due to the prolonged use of such drugs for several years, it is likely that there will be an impact on fertility.^51^

Therefore, conducting prospective large-scale studies is necessary to obtain recent, high-quality fertility data such as the FertiTOX project (www.fertitox.com) organized by FertiPROTEKT (www.fertiprotekt.com) which aims to fill the data gap on gonadotoxicity of cancer therapies to enable more accurate counseling regarding fertility preservation.^16–18^

In conclusion, this meta-analysis provides a basis for fertility counseling regarding the overall gonadal toxicity and preserved fertility after diverse CNS cancer treatments. Due to the high heterogeneity of the study population, it is not possible to provide an exact estimation of the fertility prognosis; however, the data indicate that overall gonadal toxicity is moderate (20%, 95% CI: 10%–34%). Thus, fertility preservation measures need to be discussed even in young patients due to the lack of large longitudinal data on individual treatment effects.

## Supplementary material

Supplementary material is available online at *Neuro-Oncology Practice* (https://academic.oup.com/nop/).

npae078_suppl_Supplementary_Data_S1

npae078_suppl_Supplementary_Data_S2

npae078_suppl_Supplementary_Data_S3

## Notes

List of unpublished papers: (1) Vidal A, Bora C, Jarisch A, et al. Impact on Fertility Outcomes in Survivors after Hematopoietic Stem Cell Transplantation for Benign and Malignant Hematologic Disorders: A systematic review and meta-analysis. unpublished data. 2024. (2) Pape J, Fernando J, Megaritis D, et al. Oncological treatments have limited effects on the fertility prognosis in testicular cancer: A systematic review and meta-analysis. unpublished data. 2024.
