# Supplementary material for: Long-term effects on fertility after central nervous system cancer: A systematic review and meta-analysis
Source: Neurooncol Pract. 2024 Aug 29;11(6):691–702. doi: 10.1093/nop/npae078 (PMC11567750; doi:10.1093/nop/npae078)
Supplement: npae078_suppl_Supplementary_Data_S1 [file npae078_suppl_supplementary_data_s1.docx]

**Supplementary Table and Figure Legends.**

**S1** *Database Search Strategies*

Systematic literature search in Medline, Embase and Cochrane.

**S2** *Characteristics of the included studies*

Summary of the cohort studies investigating the prevalence of gonadal toxicity and preserved fertility after CNS cancer therapies.
