# Supplementary material for: Long-term effects on fertility after central nervous system cancer: A systematic review and meta-analysis
Source: Neurooncol Pract. 2024 Aug 29;11(6):691–702. doi: 10.1093/nop/npae078 (PMC11567750; doi:10.1093/nop/npae078)
Supplement: npae078_suppl_Supplementary_Data_S2 [file npae078_suppl_supplementary_data_s2.docx]

**Database Search Strategies for Appendix**

**Research question:**
Effects of chemotherapy and radiation in brain and cns cancer patients on ovarian function and fertility

**Principal investigator:** Dr. med. Janna Pape, Frauenklinik

**Search date: February 5, 2024**

************************************************

**Ovid MEDLINE(R) ALL <1946 to February 02, 2024>**

Search Date: 05/02/2024

1 (((Brain* or cerebr* or intracran* or intra-cran* or intracerebr* or intra-cerebr* or subtentor* or sub-tentor* or supratentor* or supra-tentor* or tentor*) adj3 (Cancer* or carcin* or malign* or neoplas* or tumo?r* or leuk?emia* or metastas* or lymphoma*)) or cerebroma* or encephalophyma* or encephaloma*).ti,ab,kf. 110615

2 exp Brain Neoplasms/ 173855

3 ((Spinal cord* or spinal or spine*2 or "dura mater" or dural*3 or epidural*3 or intraspin*4 or intra-spin*4) adj3 (Tumo?r* or malign* or metastas* or neoplas* or hemangioblastoma* or hemangio-blastoma* or lipoma* or cancer*)).ti,ab,kf. 17927

4 exp Spinal Cord Neoplasms/ 11694

5 (((Glia or glial) adj3 tumo?r*) or Glioma* or ganglioglioma* or ganglio-glioma* or ganglioneuroma* or ganglio-neuroma* or gangliocytoma* or ganglio-cytoma* astrocytoma* or astro-cytoma* or oligoastrocytoma* or oligo-astrocytoma* or gliosarcoma* or glio-sarcoma* or oligodendroglioma* or oligodendro-glioma* or oligo-dendroglioma* or oligodendrocytoma* or oligodendrocytos* or (Glioblastoma* or Glio-blastoma* or glyoblastoma* or spongioblastoma*) or (astroglioma* or xanthoastrocytoma* or xantho-astrocytoma* or xanthoastro-cytoma*) or (Ependymoma* or ependymoblastoma* or ependymo-blastoma* or subependymoma* or sub-ependymoma* or (ependymal adj3 (tumo?r* or glioma*)))).ti,ab,kf. 119337

6 exp Ganglioneuroma/ 2708

7 (Medulloblastoma* or medullo-blastoma* or (blastoma* adj3 medull*)).ti,ab,kf. 9916

8 or/1-7 290972

9 (chemotherap* or chemo-therap* or chemoradiotherap* or chemo-radiotherap* or adjuvant drug therap* or carcinochemotherap* carcino-chemotherap* or antineoplastic agent* or anti-neoplastic agent* or antineoplastic drug* or anti-neoplastic drug* or antitumo?r agent* or anti-tumo?r agent* or antitumo?r drug* or anti-tumo?r drug* or anticancer* agent* or anti-cancer* agent* or anticancer* drug* or anti-cancer drug* or anticarcinogen* or anti-carcinogen* or anticancerogen* or anti-cancerogen* or ((cancer* or tumo?r* or neoplas*) adj3 treat*)).ti,ab,kf. 894929

10 exp Antineoplastic Agents/ or exp Combined Modality Therapy/ or exp Antineoplastic Combined Chemotherapy Protocols/ or exp chemotherapy, adjuvant/ or exp Antineoplastic Protocols/ or exp chemoradiotherapy/ or radioimmunotherapy/ or exp radiotherapy, adjuvant/ 1509804

11 (Radio-therap* or radiotherap* or radiationtherap* or chemoradiotherap* or radiochemotherap* or protontherap* or radiosurg* or radio-surg* or irradiation* or x-ray-therap* or therap* radiolog* or IMRT* or IORT* or radioimmunotherap* or radio-immuno-therap* or ((radiat* or irradiat* or radioisotope* or radio-isotope* or chemoradio or chemo-radio or radiochemo or radio-chemo or proton or x-ray or xray) adj2 (therap* or oncolog* or brachytherap* or brachy-therap*))).ti,ab,kf. 533715

12 exp Radiotherapy/ 209919

13 or/9-12 2349586

14 (fertili#ation* or fertility or fertile or fecund* or subfecund* or sub-fecund* or infecund* or infertility or sterility or subfert* or sub-fert* or anovularit* or gonad* or reproductive organ* or reproduction* or gamete-producing gland* or ovarian reserve* or ovary or ovaries or ovarian follicle* or oogenesis or oocyte* or amenorrhea* or premature menopaus* or early menopaus* or climacterium pr?ecox or Gonadotropin* or AMH or Anti-Mu?llerian Hormone* or Antimu?llerian Hormone* or Anti-Mu?llerian Factor* or Mu?llerian Inhibiting Hormone* or mu?llerian inhibitor* or FSH or Follicle Stimulating Hormone* or Folliculostimulating Hormone* or Follitropin or FSH-releasing hormone* or LH-FSH or testis or testes or testicle* or spermatogenes* or sperm* or semen or gametogenes* or hypogonadism* or hypo-gonadism* or "reproductive system*" or azoospermia* or spermatozoon*).ti,ab,kf. 793138

15 exp Fertility/ or exp Infertility/ or exp Gonads/ or Amenorrhea/ or anovulation/ or menopause, premature/ or Reproduction/ or Gametogenesis/ or Spermatozoa/ 381879

16 or/14-15 886085

17 8 and 13 and 16 1834

18 (exp animals/ or exp animal experimentation/ or exp models, animal/ or exp plants/ or exp fungi/) not humans/ 5631279

19 17 not 18 1760

20 limit 19 to yr="2000-current" 1107

<https://ovidsp.ovid.com/ovidweb.cgi?T=JS&NEWS=N&PAGE=main&SHAREDSEARCHID=78i6yhmRK6hbsl6elLQjknEPbbPhdozTuXzz1ivFQenEgCBEGJe2CzFDf4od0wUDH>

************************************************

**Embase <1974 to 2024 February 02>**

Search Date: 05/02/2024

1 (((Brain* or cerebr* or intracran* or intra-cran* or intracerebr* or intra-cerebr* or subtentor* or sub-tentor* or supratentor* or supra-tentor* or tentor*) adj3 (cancer* or carcin* or malign* or neoplas* or tumo?r* or leuk?emia* or metastas* or lymphoma*)) or cerebroma* or encephalophyma* or encephaloma*).ti,ab,kf. 160072

2 exp brain cancer/ 61407

3 ((Spinal cord*4 or spinal or spine*2 or "dura mater" or dural*3 or epidural*3 or intraspin*4 or intra-spin*4) adj3 (Tumo?r* or malign* or metastas* or neoplas* or hemangioblastoma* or hemangio-blastoma* or lipoma* or cancer*)).ti,ab,kf. 22695

4 exp spinal cord cancer/ 4700

5 (((Glia or glial) adj3 tumo?r*) or Glioma* or ganglioglioma* or ganglio-glioma* or ganglioneuroma* or ganglio-neuroma* or gangliocytoma* or ganglio-cytoma* astrocytoma* or astro-cytoma* or oligoastrocytoma* or oligo-astrocytoma* or gliosarcoma* or glio-sarcoma* or oligodendroglioma* or oligodendro-glioma* or oligo-dendroglioma* or oligodendrocytoma* or oligodendrocytos* or (Glioblastoma* or Glio-blastoma* or glyoblastoma* or spongioblastoma*) or (astroglioma* or xanthoastrocytoma* or xantho-astrocytoma* or xanthoastro-cytoma*) or (Ependymoma* or ependymoblastoma* or ependymo-blastoma* or subependymoma* or sub-ependymoma* or (ependymal adj3 (tumo?r* or glioma*)))).ti,ab,kf. 170058

6 exp glioma/ 180074

7 (Medulloblastoma* or medullo-blastoma* or (blastoma* adj3 medull*)).ti,ab,kf. 16557

8 exp medulloblastoma/ 19218

9 or/1-8 357006

10 (chemotherap* or chemo-therap* or chemoradiotherap* or chemo-radiotherap* or adjuvant drug therap* or carcinochemotherap* carcino-chemotherap* or antineoplastic agent* or anti-neoplastic agent* or antineoplastic drug* or anti-neoplastic drug* or antitumo?r agent* or anti-tumo?r agent* or antitumo?r drug* or anti-tumo?r drug* or anticancer* agent* or anti-cancer* agent* or anticancer* drug* or anti-cancer drug* or anticarcinogen* or anti-carcinogen* or anticancerogen* or anti-cancerogen* or ((cancer* or tumo?r* or neoplas*) adj3 treat*)).ti,ab,kf. 1350775

11 exp antineoplastic agent/ or exp multimodality cancer therapy/ or exp cancer chemotherapy/ or exp antineoplastic protocol/ 3198869

12 (Radio-therap* or radiotherap* or radiationtherap* or chemoradiotherap* or radiochemotherap* or protontherap* or radiosurg* or radio-surg* or irradiation* or x-ray-therap* or therap* radiolog* or IMRT* or IORT* or radioimmunotherap* or radio-immuno-therap* or ((radiat* or irradiat* or radioisotope* or radio-isotope* or chemoradio or chemo-radio or radiochemo or radio-chemo or proton or x-ray or xray) adj2 (therap* or oncolog* or brachytherap* or brachy-therap*))).ti,ab,kf. 734725

13 exp cancer radiotherapy/ 341910

14 or/10-13 4107177

15 (fertili#ation* or fertility or fertile or fecund* or subfecund* or sub-fecund* or infecund* or infertility or sterility or subfert* or sub-fert* or anovularit* or gonad* or reproductive organ* or reproduction* or gamete-producing gland* or ovarian reserve* or ovary or ovaries or ovarian follicle* or oogenesis or oocyte* or amenorrhea* or premature menopaus* or early menopaus* or climacterium pr?ecox or Gonadotropin* or AMH or Anti-Mu?llerian Hormone* or Antimu?llerian Hormone* or Anti-Mu?llerian Factor* or Mu?llerian Inhibiting Hormone* or mu?llerian inhibitor* or FSH or Follicle Stimulating Hormone* or Folliculostimulating Hormone* or Follitropin or FSH-releasing hormone* or LH-FSH or testis or testes or testicle* or spermatogenes* or sperm* or semen or gametogenes* or hypogonadism* or hypo-gonadism* or "reproductive system*" or azoospermia* or spermatozoon*).ti,ab,kf. 901576

16 exp fertility/ or exp infertility/ or exp semen analysis/ or exp gonad/ or exp amenorrhea/ or exp early menopause/ or reproduction/ or gametogenesis/ or spermatozoon/ 588544

17 or/15-16 1049853

18 9 and 14 and 17 2804

19 (exp animals/ or exp animal experimentation/ or exp models, animal/ or exp plants/ or exp fungi/) not humans/ 13039409

20 18 not 19 1928

21 limit 20 to yr="2000-current" 1581

<https://ovidsp.ovid.com/ovidweb.cgi?T=JS&NEWS=N&PAGE=main&SHAREDSEARCHID=3xTon52LbdFoWnaFmjP4qMRVx4qpH6AEXmLx6kHedUF1q2HoCJHHioKH7VrUxqPap>

************************************************

**Cochrane Database**

Search Date: 05/02/2024

#1 (((Brain* or cerebr* or intracran* or intra-cran* or intracerebr* or intra-cerebr* or subtentor* or sub-tentor* or supratentor* or supra-tentor* or tentor*) NEAR/3 (Cancer* or carcin* or malign* or neoplas* or tumo?r* or leuk?emia* or metastas* or lymphoma*)) or cerebroma* or encephalophyma* or encephaloma*):ti,ab,kw 8239

#2 [mh "Brain Neoplasms"] 3172

#3 (((Spinal NEXT cord*) or spinal or spine* or (dura NEXT mater) or dural* or epidural* or intraspin* or intra-spin*) NEAR/3 (Tumo?r or malign* or metastas* or neoplas* or hemangioblastoma* or hemangio-blastoma* or lipoma* or cancer*)):ti,ab,kw 780

#4 [mh "Spinal Cord Neoplasms"] 60

#5 (((Glia or glial) NEAR/3 tumo?r*) or Glioma* or ganglioglioma* or ganglio-glioma* or ganglioneuroma* or ganglio-neuroma* or gangliocytoma* or ganglio-cytoma* or astrocytoma* or astro-cytoma* or oligoastrocytoma* or oligo-astrocytoma* or gliosarcoma* or glio-sarcoma* or oligodendroglioma or oligodendro-glioma* or oligo-dendroglioma* or oligodendrocytoma* or oligodendrocytos*):ti,ab,kw or (Glioblastoma* or Glio-blastoma* or glyoblastoma* or spongioblastoma*):ti,ab,kw or (astroglioma* or xanthoastrocytoma* or xantho-astrocytoma* or xanthoastro-cytoma*):ti,ab,kw or (Ependymoma* or ependymoblastoma* or ependymo-blastoma* or subependymoma* or sub-ependymoma* or (ependymal NEAR/3 (tumo?r* or glioma*))):ti,ab,kw 4348

#6 [mh Ganglioneuroma] 1

#7 (Medulloblastoma* or medullo-blastoma* or (blastoma* NEAR/3 medull*)):ti,ab,kw 306

#8 #1 OR #2 OR #3 OR #4 OR #5 OR #6 OR #7 11627

#9 (chemotherap* or chemo-therap* or chemoradiotherap* or chemo-radiotherap* or (adjuvant NEXT drug NEXT therap*) or carcinochemotherap* carcino-chemotherap* or (antineoplastic NEXT agent*) or (anti-neoplastic NEXT agent*) or (antineoplastic NEXT drug*) or (anti-neoplastic NEXT drug*) or (antitumor NEXT agent*) or (antitumour NEXT agent*) or (anti-tumor NEXT agent*) or (anti-tumour NEXT agent*) or (antitumor NEXT drug*) or (antitumour NEXT drug*) or (anti-tumor NEXT drug*) or (anti-tumour NEXT drug*) or (anticancer* NEXT agent*) or (anti-cancer* NEXT agent*) or (anticancer* NEXT drug*) or (anti-cancer NEXT drug*) or anticarcinogen* or anti-carcinogen* or anticancerogen* or anti-cancerogen* or ((cancer* or tumor* or tumour*or neoplas*) NEAR/3 treat*)):ti,ab,kw 124032

#10 [mh "Antineoplastic Agents"] OR [mh "Combined Modality Therapy"] OR [mh "Antineoplastic Combined Chemotherapy Protocols"] OR [mh "chemotherapy, adjuvant"] OR [mh "Antineoplastic Protocols"] OR [mh "Chemoradiotherapy"] OR [mh ^Radioimmunotherapy] OR [mh "Radiotherapy, Adjuvant"] 58244

#11 (Radio-therap* or radiotherap* or radiationtherap* or chemoradiotherap* or radiochemotherap* or protontherap* or radiosurg* or radio-surg* or irradiation* or x-ray-therap* or (therap* NEXT radiolog*) or IMRT? or IORT? or radioimmunotherap* or radio-immuno-therap* or ((radiat* or irradiat* or radioisotope* or radio-isotope* or chemoradio or chemo-radio or radiochemo or radio-chemo or proton or x-ray or xray) NEAR/2 (therap* or oncolog* or brachytherap* or brachy-therap*))):ti,ab,kw 52982

#12 [mh Radiotherapy] 9961

#13 #9 OR #10 OR #11 OR #12 164493

#14 (fertilization* or fertilisation* or fertility or fertile or fecund* or subfecund* or sub-fecund* or infecund* or infertility or sterility or subfert* or sub-fert* or anovularit* or gonad* or (reproductive NEXT organ*) or reproduction* or (gamete-producing NEXT gland*) or (ovarian NEXT reserve*) or ovary or ovaries or (ovarian NEXT follicle*) or oogenesis or oocyte* or amenorrhea* or (premature NEXT menopaus*) or (early NEXT menopaus*) or (climacterium NEXT praecox) or (climacterium NEXT precox) or Gonadotropin* or AMH or (Anti-Mullerian NEXT Hormone*) or (Anti-Muellerian NEXT Hormone*) or (Antimullerian NEXT Hormone*) or (Antimuellerian NEXT Hormone*) or (Anti-Mullerian NEXT Factor*) oe (Anti-Muellerian NEXT Factor*) or (Mullerian NEXT Inhibiting NEXT Hormone*) or (Muellerian NEXT Inhibiting NEXT Hormone*) or (mullerian NEXT inhibitor*) or (muellerian NEXT inhibitor*) or FSH or (Follicle NEXT Stimulating NEXT Hormone*) or (Folliculostimulating NEXT Hormone*) or Follitropin or (FSH-releasing NEXT hormone*) or LH-FSH or testis or testes or testicle? or spermatogenes* or sperm* or semen or gametogenes* or hypogonadism* or hypo-gonadism* or (reproductive NEXT system*) or azoospermia* or spermatozoon*):ti,ab,kw 43654

#15 [mh Fertility] OR [mh Infertility] OR [mh Gonads] OR [mh ^Amenorrhea] OR [mh ^anovulation] OR [mh ^"menopause, premature"] OR [mh ^Reproduction] OR [mh ^Gametogenesis] OR [mh ^Spermatozoa] 7214

#16 #14 OR #15 43726

#17 #8 AND #13 AND #16 with Cochrane Library publication date Between Jan 2000 and Feb 2024 118

<https://www.cochranelibrary.com/advanced-search/search-manager?search=7376288>
