# Supplementary material for: Long-term effects on fertility after central nervous system cancer: A systematic review and meta-analysis
Source: Neurooncol Pract. 2024 Aug 29;11(6):691–702. doi: 10.1093/nop/npae078 (PMC11567750; doi:10.1093/nop/npae078)
Supplement: npae078_suppl_Supplementary_Data_S3 [file npae078_suppl_supplementary_data_s3.docx]

| **First author, year of publication**  **Supplement 2** **Characteristics of the included studies** | **Country** | **Study design** | **Number of patients (treatment period)** | **Age at diagnosis / therapy**  **(yrs, mean ± SD or range)** | **Age at outcome / evaluation**  **(yrs, mean ± SD or range)** | **Follow-up (yrs, mean ± SD or range)** | **Tumor type (%), details** | **Surgery (%), details** | **Chemotherapy (CT) (%), details** | **Radiation (RT) (%), details** | **Fertility parameters pre-treatment** | **Gonadal toxicity (%)** | **Preserved fertility*** **post-treatment (%)** |
| --- | --- | --- | --- | --- | --- | --- | --- | --- | --- | --- | --- | --- | --- |
| Saeki et al., 2000 | Japan | retro-spective | 16 | not reported | At admission: 19.9 (17-26, women),  12.4 (6-24, men) | 7.9 (1.2-16.4) | Germinoma 16 (100), Location: suprasellar 10 (62.5), sellar 6 (37.5) | 9 (56.3) | 8 (50),  standard | 16 (100),  30-50 Gy | not reported | not reported | 7/16 (43.5)  (no hypogonadism) |
| Gurney et al., 2003 | USA | retro-spective, survey | 1607  (1970-1986) | At diagnosis:  < 20 | At survey:  up to 39  (98.8% of cohort) | at least 5 years from diagnosis | Ependymal: 118 (7.3)  Glioma: 1066 (66.3)  Medulloblastoma/PNET: 343 (21.3)  Others: 80 (5.0) | 1542 (96.0), with RT and/or CT | 446 (27.8)  (surgery + CT + RT) | 682 (42.4) (surgery + RT), dose brain:  40-55 Gy,  dose spine:  <10-35 Gy | not reported | not reported | 1535/1607 (95.5)  (no medication to induce puberty 5 years postdiagnosis to survey) |
| Agha et al., 2005 | Ireland | case-control | 76:  56 (case + RT), 20 (- RT) | At RT:  33.3 ± 12.0 | At evaluation: 39.3 ± 10.1 | 3.16 (1-12.5) | Glioma: 59 (77.6) Medulloblastoma: 2 (2.6)  Meningioma: 6 (7.9)  Others: 9 (11.8) | 49 (64.5), Biopsy only: 25 (32.9) | 16 (21.1),  standard | 56 (73.7),  External beam 54 (4-97) Gy | not reported | 4/76 (5.3)  (Primary testicular failure after CT) | 61/76 (80.3)  (no gonadotropin deficiency or panhypopituitarism) |
| Cuny et al., 2011 | France | retro-spective | 56, prepubertal (1989-2009) | At therapy: boys <13,  girls <11 | boys >13,  girls >11 | not described | Ependymal: 11 (19.6)  Medulloblastoma: 45 (80.4) | 56 (100), 3 with ovarian transposition before RT | 38 (67.9),  standard //  stem cell transplant: 6 (10.7) | 56 (100), cranial and spinal RT, dose 5-49 Gy | not reported | 12/56 (21.4)  (Tubular or testicular deficiency / Primary ovarian failure) | 37/56 (66.1) (no gonadal toxicity or hypogonadotropic hypogonadism) |
| Madaschi et al., 2011 | Italy | case-control | 32,  26 (case, hypothalamic tumor),  6 (extrasellar)  (2005 - 2009) | At diagnosis: cases: 38.5 (33-47), controls: 35.5 (31.5-44) | not reported | 5 years | Glioma: 29 (90.6), Meningiomas 2 (6.3),  Others: 10 (3.1) | 26 (81.3),  Total: 13 (40.6), Partial:  12 (37.5),  Biopsy only / none: 7 (21.9) | 12 (37.5),  standard | 26 (81.3) fractionated external, dose to hypothalamic region: 41.8 Gy (30.7-49.8) | not reported | not reported | 22/23* (95.7)  *27 tested, excluding 4 menopausal women  (no gonadotropin deficiency) |
| Preusser et al., 2011 | Austria | cross-sectional | 11 women | not reported | 35 (28-40) | 3.3 (1-10.6) (Time between diagnosis / hormonal assessment) | Glioma: 11 (100) | 11 (100),  total resection | Surgery + CT + RT: 11 (100),  standard CT | Conformal brain radiation up to 66 Gy (54-66) | not reported | 4/11 (36.4)  (Menopausal hormone pattern) | 7/11 (63.6)  (no menopausal pattern) |
| Shalitin et al., 2011 | Israel | retro-spective | 114, 65 prepubertal (1986-2005) | 7.07 ± 5.42 | 15.57 (3.8-30) | 12.8 (3.7-28.7) | Ependymal: 9 (7.9)  Germ cell: 6 (5.3)  Glioma: 56 (49.1)  Medulloblastoma: 29 (25.4)  Others: 14 (12.3) | 92 (80.7),  Brain: 90 (79.0)  Spinal: 2 (1.8) | 78 (68.4),  stem cell transplant: 5 (4.4) | 55 (48.2)  CT + RT: 3 (2.6) Surgery + CT + RT: 52 (45.6) | not reported | 3/114 (2.6)  (Primary gonadal failure, all pre-pubertal) | 105/114 (92.1) (no gonadal failure or hypogonadotropic hypogonadism) |
| Viswana-than et al., 2011 | USA | retro-spective | 31  (2000-2008) | ≤18 years | 11.9 ± 3.3 | 1.8 ± 0.8 | Ependymal: 2 (6.5)  Glioma: 3 (9.8)  Medulloblastoma: 6 (19.4)  Sellar: 4 (12.9)  Others: 11 (35.5) | 28 (90) | 22 (71.0),  CT not specified | 31 (100),  proton beam + conventional RT | not reported | not reported | 27/31 (87.1)  (no hypogonadotropic hypogonadism) |
| Koustenis et al., 2013 | Germany | survey | 203 | 11 (1-15) | 22 (19-27) | 25 (19-43) | Ependymal: 6 (3)  Germ cell: 55 (27.1)  Glioma: 62 (30.7)  Medulloblastoma: 68 (33.5)  Others: 10 (4.9) | not specified | 118 (58.1) | 152 (74.9), local RT + 118 with craniospinal RT, dose: 0-17, 18-29, ≥30 Gy | Infertility diagnosis:  10 (4.9), all males | 18/203 (8.8)  (Azoospermia 6, Oligozoospermia 12) | 171/203 (84.2)  (no gonadal toxicity or amenorrhea) |
| DeWire et al., 2014 | USA | pros-pective | 30,  women only (1996-2003) | At diagnosis: 6.7 (3.0–18.0) | Last evaluation:  14.8 (9.4–26.8) | 7.1 (4.0–10.8) | Embryonal tumor (100%) | 30 (100) | 30 (100), high-dose // stem cell transplant 30 (100) | 30 (100), photon RT, craniospinal (risk-adapted) | not reported | 16/29 (55.2)  (POI, FSH > 15 or FSH > 25, dependent of Tanner stage) | 13/29 (44.8)  (no POI at end of follow-up) |
| Balachan-dar et al., 2015 | USA | retro-spective | 31,  women only  (1980-2008) | 7.7 (1.6-17.8) | 16.6 (10.2-32.6) | 9 | Medulloblastoma: 31 (100) | 31 (100) | 31 (100),  standard 29 (93.5),  high dose 2 (6.5) //  stem cell transplant: 5 (16.1) | 29 (93.5), photon/proton RT, dose: 26-28 Gy (low risk), 37.8-42 Gy (high risk), dose to the ovaries ca. 5% | not reported | 6/31 (19.4)  (Primary ovarian failure) | 25/31 (80.6)  (no ovarian failure) |
| Pfitzer et al., 2014 | Germany | retro-spective | 144  (2000-2005) | At diagnosis:  7 (3-11) | 18  (81% > 13 years) | not reported | Ependymal: 9 (6.3)  Glioma: 84 (58.3)  Medulloblastoma: 34 (23.6) Meningioma: 1 (0.7)  Others: 17 (11.9) | 48 (33.3) | 85 (59.0),  standard 62 (43.1),  gonadotoxic 23 (16) | 42 (29.2),  > or < 30 Gy | not reported | 14/41 (34.1)  (FSH ≥10 IU/l and subsequent decrease of hormone values) | 40/41 (97.6)  (no fertility after 11-12 years since diagnosis only in 1 case = 2.4%) |
| Shih et al., 2014 | USA | pros-pective | 20  (2007-2010) | 37.5 (22-56) | not reported | 5.0 (3.3-5.9) | Glioma: 20 (100  Location: Frontal: 9 (45), (fronto)temporal 8 (40), parietal 2 (10), Occipital 1 (5) | 16 (80)  Partial: 12 (60), Total: 4 (20), Biopsy only: 4 (20) | 18 (90) | 20 (100),  proton RT,  dose 54 Gy, 30 fractions | 0 (0) (Gonadal deficits) | not reported | 18/20 (90)  (no central hypogonadism) |
| Uday et al., 2015 | UK | cross-sectional | 35  (1982-2002) | At diagnosis:  8 (2-14)  At end of treatment:  9 (4–15) | 23 (16-35) | 18 (10-28) | Medulloblastoma: 35 (100) | 35 (100) | 23 (65.7),  standard | 35 (100), Conventional photon RT,  total dose 55 Gy (94.3% of cases) | not reported | not reported | 27/35 (71.1)  (no hypogonadism) |
| Pietila et al., 2017 | Finnland | cross-sectional | 52, 16 prepubertal (1983-1997) | At diagnosis:  6.0 (0.1-15.5)  At start of RT: 7.2 (0.2-20.9) | 14.2 (3.8-28.7) | 7.5 (1.5-15.1) | Glioma: 52 (100),  Location: Infratentorial 25 (48), Supratentorial 27 (52), Brain stem 5 (10), Cerebral hemisphere 13 (25), (Para)sellar 7 (13), Pituitary 1 (2), Hypothalamus: 5 (10), Optic chiasma: 1 (2) | 29 (56) | 17 (33),  standard,  CT + RT: 14 (27) | 20 (38), incl. hypothalamo–pituitary axis (19/24 = 79.2%) | not reported | not reported | 44/52 (84.6)  (no sex hormone deficiency) |
| Vatner et al., 2018 | USA | pro-spective | 222,  189 included in analysis (2003-2016) | At treatment: 7.4 (1.1-25.9) | not reported | 4.4 (0.1-13.3) | Ependymal: 26 (13.8)  Glioma: 19 (10)  Medulloblastoma: 130 (68.8)  Meningioma: 3 (1.6)  Sellar: 2 (1.1)  Others: 9 (4.8) | 171 (90.5),  Total: 124 (65.6), Subtotal: 19 (10.1), Partial:  28 (14.8),  Biopsy: 9 (4.8), un-known: 9 (4.8) | 189 (100),  CT not specified | 133 (70.4),  Proton craniospinal + boost, Hypothalamus dose: 26.0 (0-54.6),  Pituitary dose:  24.3 (0-57.3) | Baseline endocrino-pathy:  31 (16.4) | not reported | 58/61 (95)  (no gonadotropin deficiency after 6 years) |
| Jalali et al., 2019 | India | pro-spective | 51 | 13.5 (5-25) | not specified | 5.9 (2-12.5) | Glioma: 7 (14.2)  Sellar: 15 (29.4)  Others: 10 (19.6) | 51 (100) debulking,  all combined with RT | 0 (0) | 51 (100), stereotactic RT, 54 Gy in 30 fractions | HPG dysfunction:  8 (16) | not reported | 40/51 (78.4)  (no HPG dysfunction) |
| Santos et al., 2019 | Spain | retro-spective (1997-2016) | 23, prepubertal  (1997-2016) | 3-12 | 18 | 5-13.5 | Medulloblastoma: 23 (100) | 15 (65.2),  total resection | numbers not specified,  stem cell transplant:  8 (34.8) | numbers not specified,  dose of cranial / craniospinal RT: 55.7 / 29.7 Gy | not reported | 1/23 (4.3)  (Hypergonadotropic hypogonadism) | 19/23 (82.6)  (no hyper-/ hypogonadotropic hypogonadism) |
| Van Iersel et al., 2020 | USA | retro-spective | 355, 254 (71.5%) with hormonal analysis (1996-2016) | At diagnosis: 4.6 (0.2-24.6), At treatment-beginning: 6.4 (0.9-24.9) At RT: 13.2 (6.5-19.1) | 17.76 (2.0-40.5) | 10.1 (0.1-19.6) | Ependymal: 193 (54.4)  Glioma: 162 (45.6), | 276 (77.75), combined with RT | 125 (35.2), standard,  combined with RT | 355 (100), photon RT,  dose 50.4-59.4 Gy | not reported | not reported | 209/254 (82.3)  (no LH/FSH deficiencies) |
| Xiang et al., 2020 | China | retro-spective | 77  (2010-2017) | 15 (12.5-17) | At last visit:  18 (16-20) | Endocrine follow-up: 1.6 (0.33-3.5) | Germ cell: 77 (100) Location: (Supra)sellar 51 (66.2) | 13 (16.9),  Total: 6 (19.4), Subtotal: 1 (1.3), Partial: 4 (12.9), Biopsy: 8 (10.4) | 31 (40.3),  standard, in combination with RT | 77 (100), conventional fractionated RT, Hypothalamus dose: 49.4 Gy (25.8-53.8), Pituitary dose: 46.2 Gy (24.8-51.9) | not reported | not reported | 33/76 (43.4)  (no central hypogonadism) |
| Haghiri et al., 2021 | France | retro-spective | 145 | At diagnosis: 2.6 (0-18.2) | At last visit:  18.3 (6.8-43.1) | 15 (5-34) | Neuroblastoma: 145 (100) | 142 (97.9) | 145 (100), high dose for stem cell transplantation | 66 (45.5), Primary site: 27 Gy (19-40),  Metastatic sites: 30 Gy, (20-40) | not reported | 88/105 (83.8)  (Ovarian failure, Testicular failure, n=73 after busulfan) | 17/105 (16.2)  (no gonadal insufficiency) |
| Maciel et al., 2021 | Portugal | retro-spective | 242  (1994 - 2018) | At diagnosis: 6.7 (0–18) | At first endocrine evaluation:  9.6 (1.5-19.2) | 9.8  (0.4-31.2) | Ependymal: 37 (15.3)  Germ cell: 20 (8.3)  Glioma: 81 (33.5)  Medulloblastoma: 65 (26.9)  Location: Posterior fossa 93 (38.4), sellar /suprasellar 70 (28.9) | 193 (79.8) | 184 (76.0),  standard | 242 (100), intensity modulated  Cranial dose:  54 Gy (24-60), Spinal dose:  30.6 Gy (23.4-39.6) | not reported | not reported | 181/242 (74.8)  (no hypogonadism) |
| Zhang et al., 2021 | China | case-control | 127, 75 (59.1%) with follow up, 29 prepubertal (2006-2019) | 14.6 (9.3-20.1) | Prepubertal: 13 years for girls, 14 years boys, Postpubertal:  no data | 4.25 (2.6 - 5.3) | Germinoma: 73 (57.5)  No histology: 54 (42.5)  Location: Suprasellar 127 (100) | 13/105 (12.4) | 60/105 (57.1)  CT + RT: 54/105 (51.4) Surgery + CT + RT: 6/105 (5.7) | 31/105 (29.5) | HPG-dysfunction: 64/75 (85.3) | not reported | 29/46 (63.0)  (recovery of HPG dysfunction) |
| Gonzales et al., 2022 | France | pros-pective | 221  (2010-2015) | 6.7 (0-15.9) | 8.5 (1-17.4) | 15.6 (3.5-30.9) | Germ cell: 9 (4)  Glioma: 45 (20.5)  Medulloblastoma: 83 (37)  Sellar: 64 (29)  Others: 20 (9) | 199 (90) | 125 (56.6),  low to high-risk CT | 174 (78.7), photon RT,  dose suprasellar 52 (24-70) Gy, otherwise 30.2 (0-68) | not reported | 37/221 (16.7) (Gonadal toxicity) | 142/221 (64.3)  (no gonadal toxicity or hypogonadotropic hypogonadism |
| Margolis et al., 2022 | Israel | retro-spective | 59 | 2.85 (0.7-7.2) | 3 (1-5) | at least 1 year | Glioma: 59 (100)  Location: Optic tract | 18 (30.5), debulking | 30 (50.8),  biologicals 12 (20.3) | 4 (6.8) | not reported | 2/52 (3.8)  (Primary gonadal failure) | 45/52 (86.5) |
| Merchant et al., 2022 | USA | pros-pective | 101 | At diagnosis: 7.77  (1.29–17.52)  At treatment-beginning:  8.98  (3.20–17.63) | 10 | 14.94  (7.23–21.5) | Craniopharyngioma:  101 (100) | 101 (100), mainly limited | 0 (100) | 101 (100), photon RT (conformal or intensity-modulated) | LH/FSH deficiency  2/101 (2) | not reported | 37/99 (37.4)  (no LH/FSH deficiency) |
| Partenope et al., 2022 | USA | retro-spective | 55  (1996-2016) | 11.6 (5.4-18.3) | 17.9 (1.6-36.8) | 6.57 (0.4-20.8) | Germ cell: 55 (100) | 54 (98.2) | 42 (76.4),  standard //  stem cell transplant: 7 (12.7) | 50 (90.9),  whole ventricular field RT with boost: 21/50 (42%) | HPG dysfunction:  6 (10.7) / Arrested puberty:  3 (5.5) | not reported | 32/52* (61.5)  (*excl. patients with pre-treatment HPG dysfunction)  (no low gonadotropin levels) |
| Abali et al., 2023 | Turkey | retro-spective | 65, girls | At diagnosis:  7.4 ± 3.6  (0.7-16.9) | At admission:  8.7 ± 3.6  (1.0-17.1) | 7.4 (2.2-13.4) | Medulloblastoma: 17 (26.2)  Craniopharyngioma: 29 (44.6)  Others: 19 (29.2) | 65 (100), 2 with residual tumor | 23 (35.4), combined with RT | 37 (56.9),  cranial (27.7) or craniospinal (29.2) | not reported | 3/65 (4.6) (Hypergonadotropic hypogonadism) | 42/65 (64.4)  (no hyper- or hypogonadotropic hypogonadism) |
| Merchant et al., 2023 | USA | retro-spective | 156 (100 males, 56 females) (2003-2013) | At treatment: 8.54  (3.21-21.66) | not reported | 11.08  (0.58-16.46) | Medulloblastoma:  156 (100) | 156 (100), gross-total resection | 156 (100),  high-dose | 156 (100), craniospinal + boost, photon RT (conformal or intensity modulated), high-risk (36-39.6 Gy), standard risk (23.4 Gy) | not reported | 21/33 (63.6)  (males: azoospermia, females: low AMH) | 39/156 (25.0)  (no hypogonadism after 10 years) |
| Rosimont et al., 2023 | France | retro-spective | 204  (2010-2015) | Girls:  6.5 (0-15.1)  Boys:  7.7 (0.1-15.9) | not reported | mean: 8.4 (girls), 10 (boys) | Germinoma: 8 (3.9)  Glioma: 44 (21.6)  Medulloblastoma: 71 (34.8) | 182 (89.2) | 83 (40.7),  low, moderate, high-risk | 15 (7.4), conventional RT versus proton therapy | not reported | 50/186 (26.7)  (Gonadal toxicity) | 86/136 (63.2)  (no gonadal toxicity) |
| Stern et al., 2023 | Israel | retro-spective | 62,  (1987-2021) | At diagnosis:  8 (1-29) | At last visit:  17.5 (7-40) | 10.6 (3-28) | Medulloblastoma: 62 (100) | 62 (100), resection,  6 cases with ovarian tissue transplants | 60 (96.8),  standard protocols | 59 (95.1),  craniospinal + boost  high-risk (36 Gy), standard risk (23.4 Gy) | not reported | 47/62 (76.0)  (elevated FSH, low AMH) | 15/62 (24.2)  (no gonadal toxicity) |

* Def.: Basal LH or FSH levels above the upper limit of the reference range and/or low AMH levels in women or low inhibin B in men and/or azoo-/oligospermia

** Def.: No signs of gonadal toxicity incl. primary / secondary amenorrhea, no central / primary / secondary hypogonadism or panhypopituitarism
